# Campylobacteriosis Outbreak Linked to Municipal Water, Nebraska, USA, 2021^1^

**DOI:** 10.3201/eid3010.231509

**Published:** 2024-10

**Authors:** Lauren Jansen, Rachael Birn, Samir Koirala, Sadie Oppegard, Brianna Loeck, Jeff Hamik, Elizabeth Wyckoff, Dana Spindola, Sue Dempsey, Amanda Bartling, Alexis Roundtree, Amy Kahler, Charlotte Lane, Nancy Hogan, Nancy Strockbine, Haley McKeel, Jonathan Yoder, Mia Mattioli, Matthew Donahue, Bryan Buss

**Affiliations:** Nebraska Department of Health and Human Services, Lincoln, Nebraska, USA (L. Jansen, R. Birn, S. Koirala, S. Oppegard, B. Loeck, J. Hamik, E. Wyckoff, M. Donahue, B. Buss);; Centers for Disease Control and Prevention, Atlanta, Georgia, USA (L. Jansen, E. Wyckoff, A. Kahler, C. Lane, N. Hogan, N. Strockbine, H. McKeel, J. Yoder, M. Mattioli, B. Buss);; Council of State and Territorial Epidemiologists, Atlanta (R. Birn); University of Nebraska–Lincoln, Lincoln (S. Koirala, B. Loeck, J. Hamik);; East Central District Health Department, Columbus, Nebraska, USA (D. Spindola);; University of Nebraska Medical Center, Omaha, Nebraska, USA (S. Dempsey, A. Bartling);; Chenega Enterprise System and Solutions, Chesapeake, Virginia, USA (A. Roundtree);; US Public Health Service, Lincoln (B. Buss)

**Keywords:** bacteria, water, Campylobacter infections, Campylobacteriosis, outbreaks, Nebraska, United States

## Abstract

In September 2021, eight campylobacteriosis cases were identified in a town in Nebraska, USA. We assessed potential exposures for a case–control analysis. We conducted whole-genome sequencing on *Campylobacter* isolates from patients’ stool specimens. We collected large-volume dead-end ultrafiltration water samples for *Campylobacter* and microbial source tracking testing at the Centers for Disease Control and Prevention. We identified 64 cases in 2 waves of illnesses. Untreated municipal tap water consumption was strongly associated with illness (wave 1 odds ratio 15.36; wave 2 odds ratio 16.11). Whole-genome sequencing of 12 isolates identified 2 distinct *Campylobacter jejuni* subtypes (1 subtype/wave). The town began water chlorination, after which water testing detected coliforms. One dead-end ultrafiltration sample yielded nonculturable *Campylobacter* and avian-specific fecal rRNA genomic material. Our investigation implicated contaminated, untreated, municipal water as the source. Results of microbial source tracking supported mitigation with continued water chlorination. No further campylobacteriosis cases attributable to water were reported.

*Campylobacter* is a gram-negative, microaerophilic, flagellated, helical bacterium (*1*). *Campylobacter* infection, or campylobacteriosis, is the most common bacterial cause of diarrhea in the United States, producing ≈1.5 million illnesses each year (*2*). Serious sequelae of infection can occur, including prolonged bowel symptoms (5%–20% of reported cases), reactive arthritis (1%–5% of reported cases), and Guillain-Barré syndrome (0.001% of reported cases) (*2*). Common causes of *Campylobacter* infection include consuming contaminated foods (especially poultry, meat, and dairy) or contaminated water or having contact with animals (*3*). Campylobacteriosis outbreaks associated with water have been linked to contamination of groundwater sources after heavy rainfall and problematic water system cross-connections (*4*,*5*).

In Nebraska, USA, incidence of campylobacteriosis increased from 599 cases/year in 2016 to 811 cases/year in 2019 (*6*,*7*). This observed increase coincided with an increased use of culture-independent diagnostic methods. As of 2015, Nebraska had the second-highest incidence of campylobacteriosis in the United States, at 26.6 cases/100,000 population, second only to South Dakota (*8*). Given competing priorities for public health, particularly during the COVID-19 pandemic, investigation of campylobacteriosis cases is sometimes limited to application of case definitions and assignment of case status, consistent with the National Notifiable Disease Surveillance System designation (*9*). Most cases are classified as sporadic infections. Selected cases are further investigated by local or state public health authorities, particularly if spatial or temporal clustering is observed.

Nondisinfected drinking water systems are common in the United States; >20 million US residents were served by nondisinfected water in 2006 (*10*). Nebraska is 1 of 6 states where ground water is used for >75% of public water supplies (*11*). In most Nebraska communities, ground water supplies are not treated with a disinfectant before being supplied to the public (*12*). Under the US Environmental Protection Agency (EPA) Ground Water Rule, ground water sourced systems can be untreated if certain monitoring procedures, which depend on the population size, are followed (*13*,*14*).

Contamination of drinking water systems can occur by many routes. One study reported contamination by animal feces and wastewater to be the most common source of contamination in ground water systems (*15*). Microbial source tracking (MST) is a molecular tool used to identify sources of fecal pollution in water. This technique is specialized and is not part of routine ground water monitoring procedures. MST uses quantitative PCR to detect molecular evidence of microbes unique to the guts of humans or specific animal species (*16*,*17*). MST assays have been developed to identify multiple species in the United States, including humans, cattle, and birds (*18*–*20*). Moreover, recently EPA and the National Institute for Standards validated standard material for MST biological markers, enabling use of these MST methods in outbreak response (*21*).

In September 2021, the East Central District Health Department and Nebraska Department of Health and Human Services were notified by electronic laboratory reporting of 8 campylobacteriosis cases clustering in 1 week’s time in a small town in east central Nebraska that had a population of ≈330 persons. During 2011–2020, median annual campylobacteriosis case count in the affected town was 1 (range 0–8). The Nebraska Department of Health and Human Services collaborated with the East Central District Health Department to investigate, find the exposure and contamination sources, and prevent additional illness. In this report, we describe this outbreak of campylobacteriosis that was linked to municipal drinking water and animal contamination and identified by using MST.

## Methods

### Epidemiologic Investigation Methods

In response to the cluster of 8 campylobacteriosis cases, we sought to find additional cases. We used the case definitions from the 2015 National Notifiable Disease Surveillance System case definition for campylobacteriosis, in which a probable case is defined as “a case that meets the probable laboratory criteria for diagnosis or a clinically compatible case that is epidemiologically linked to a probable or confirmed case of campylobacteriosis” and a confirmed case is defined as “a case that meets the confirmed laboratory criteria for diagnosis” (*22*). We created a broad hypothesis-generating questionnaire in REDCap (a web-based interface for secure data collection; https://www.project-redcap.org) to address consumption of meats and other food items; consumption of different water sources, including methods of in-home water treatment (e.g., filtration, reverse osmosis); attendance at 2 known large community gatherings (i.e., a town bazaar and a wedding); and recent animal contact. We sought to reach as many town residents, workers, and visitors as possible with this questionnaire, including those sickened during the outbreak and those who remained healthy. To solicit participation among community members, the questionnaire link was distributed using Facebook (Meta Platforms, https://www.meta.com) posts by willing town residents and the local health jurisdiction’s Facebook page. We deployed the questionnaire on September 16, 2021. On the basis of preliminary results of this initial questionnaire, which included multiple, free-text responses noting consumption of home-grown produce, we developed a second supplemental questionnaire through REDCap. By using respondent emails collected in the initial questionnaire, we deployed the website for this second questionnaire on September 28, 2021, to address produce consumption, with a particular focus on locally grown items and home gardens. If respondents to the first questionnaire did not respond to the second questionnaire but had previously provided a telephone number, we made attempts to complete the second questionnaire with the resident by telephone. Finally, both questionnaires were administered during door-to-door canvassing in the affected community, with 6 investigators working in pairs to conduct in-person interviews with residents. We obtained verbal consent from each adult or legal guardian before administering interview questionnaires. We did not document refusal to participate. 

This activity was reviewed by CDC and was conducted consistent with applicable federal law and CDC policy (e.g., 45 C.F.R. part 46, 21 C.F.R. part 56; 42 U.S.C. §241(d); 5 U.S.C. §552a; 44 U.S.C. §3501 et seq.). This activity was determined to be public health surveillance and therefore did not require CDC Human Research Protection Office review. We completed data collection through both questionnaires on October 21, 2021. We also obtained data from Nebraska’s Electronic Disease Surveillance System (NEDSS), particularly in cases when a person could not be reached for an interview. Given the potential for nonresidents to have been exposed in the affected town, we also used NEDSS to search for other campylobacteriosis cases occurring within bordering counties during the outbreak, from mid-August 2021 to October 2021, to identify additional cases potentially associated with the outbreak. 

Where possible, we combined results of the 2 questionnaires (or NEDSS data if questionnaire data were unavailable) to create 1 entry per person sick with campylobacteriosis. We used questionnaire results for a case-control analysis. We defined probable or suspect cases as self-reported diarrheal illness (>3 stools/24 h for probable cases; 1–2 stools/24 h for suspect cases) among town residents, or among workers or visitors with exposure in the town, with onset during August 30–October 8, 2021; confirmed cases had *Campylobacter* spp. detected in stool (e.g., by antigen testing, PCR, or culture). Controls were persons who denied any diarrheal illness. We did not conduct any matching. If *Campylobacter* culture isolates were obtained from a specimen, whole-genome sequencing (WGS) was performed by the Nebraska Public Health Laboratory using the MagNA Pure Compact Nucleic Acid Isolation Kit version 19 (Roche, https://lifescience.roche.com) (*23*–*27*). We submitted sequences to GenBank.

We extracted data from REDCap by using Excel (Microsoft, https://www.microsoft.com) and performed descriptive analysis and calculated odds ratios (ORs) by using CDC’s EpiInfo 7.2.4.0 (https://www.cdc.gov/epiinfo). We conducted analyses of questionnaire results and conducted separate subanalyses for each wave of illness. We calculated 95% CIs by using OpenEpi version 3.01 (https://www.openepi.com). We applied the modified Haldane–Anscombe correction in a case of a zero cell. We classified users of water-filtering pitchers or refrigerator water filters as being exposed to untreated water and those using reverse osmosis as being unexposed to untreated water.

### Environmental Investigation Methods

Epidemiologic investigation findings guided the environmental investigation that followed outbreak detection. The town’s ground water wells were routinely tested monthly for coliform. In Nebraska, water operators must be licensed and complete continuing education, and all public water testing results are documented in the Safe Drinking Water Information System database and are available to the public (*28*). A 100-mL sample was collected by the town water operator at a predetermined site in accordance with the water system’s approved sample site plan. Testing was conducted by the state laboratory. When coliforms were detected, repeat samples were collected from the original site and locations upstream and downstream of the original site. Trigger samples (i.e., samples collected in response to coliform detection) were also collected from groundwater sources after a routine coliform detection. Routine maintenance chlorination of the system, typically done in spring and fall each year, was begun on October 1, 2021.

While the outbreak investigation was ongoing, the Nebraska Department of Environment and Energy (NDEE) began a comprehensive evaluation of the municipal water system. NDEE conducted an external inspection of the town’s water tower by using an NDEE drone on October 18, 2021. NDEE drinking water field representatives performed a walkthrough backflow inspection in the town on October 20, 2021. When routine maintenance chlorination was stopped, postchlorination water testing was carried out on October 26 and October 29, 2021; positive results prompted NDEE to issue a boil water advisory on October 30, 2021, and to request specialized *Campylobacter* water testing from CDC.

On November 4, 2021, a total of 6 large-volume (>100-L), dead-end ultrafiltration (DEUF) water samples were collected by NDEE by using methods described previously (*29*). Sampled locations included the 2 active town wells and 4 additional sites from the distribution system in the town that we selected on the basis of previous coliform bacteria detections. We conducted sampling in areas within the distribution system with recently positive total coliform testing or in areas of concern, including areas with potential cross-connections, suspected backflow, dead ends, or high water age (i.e., the time water spends within the distribution system before use) (Appendix Table 1). After sample collection, water chlorination resumed. We submitted all DEUF samples to CDC for *Campylobacter*-specific DNA testing and culture. In addition, CDC performed MST by using previously described PCR methods to identify bacteria molecular markers unique to human, ruminant, and bird feces (known environmental fecal shedders of *Campylobacter*) (*18*–*20*).

## Results

### Epidemiologic Investigation Results

A total of 138 questionnaires were completed. Of those, 129 were completed by persons connected to the affected community (121 town residents [37% of the town’s population] and 8 with exposure in the community but not residing there). Of the 129 questionnaires, 26% (34/129) were completed during door-to-door canvassing efforts and the remainder by Facebook recruitment, email, or telephone (percentage completed by each method was not captured). According to US Census American Community Survey data, the median age of town residents was 36.8 years, and 55% identified as female in 2021 (*30*). By comparison, the median age of the 129 community-linked questionnaire respondents was 56 years, and 63% were female. 

We identified 64 total campylobacteriosis cases (21 confirmed, 38 probable, and 5 suspect). Ill persons had a median age of 56 years (range 2–90 years); 33 (52%) were female and 31 (48%) male. Seven (11%) persons reported hospitalization (median length of stay 1 night [range 1–3 nights]), and no deaths occurred. Ill persons experienced diarrhea (64/64, as described in the case definition), abdominal cramping (48/64), nausea (34/64), fever (33/64), and vomiting (12/64).

We identified 2 distinct waves of illness; the first occurred during August 30–September 15 and the second during September 29–October 8 (Figure). Peak reported illness onset was during or shortly after the 2021 Labor Day holiday weekend (September 4–6, 2021). For wave 1, all but 1 (48/49 [98%]) of ill persons reported municipal water exposure, compared with 76% (25/33) of controls (OR 15.36 [95% CI 1.82–129.82]). For wave 2, all 14 (100%) ill persons reported municipal water exposure, compared with 65% (22/34) of controls (OR undefined). To account for no unexposed ill persons, we applied a modified Haldane–Anscombe correction, which yielded an OR of 16.11 (95% CI 0.88–293.6). Among all other exposures analyzed, including 50 separate food items, contact with animals or manure, and attendance at a community gathering, we found no other statistically significant associations with illness (Appendix Tables 2, 3).

**Figure:**
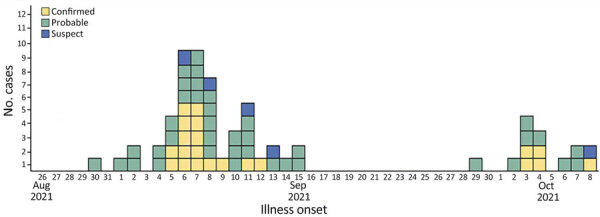
Number of confirmed, probable, and suspect campylobacteriosis cases (N = 64) in an outbreak linked to municipal water, by illness onset date, Nebraska, USA, August–October 2021.

Among 21 cases confirmed by laboratory testing, 8 were by antigen testing only, 1 by PCR, and 12 by stool culture. We speciated and identified all 12 clinical isolates as *C. jejuni*. WGS of 12 *Campylobacter* isolates from 12 patients’ stool specimens identified 2 distinct *C. jejuni* subtypes, with 1 subtype/wave. In wave 1, a total of 7 isolates from 7 persons in 6 separate households were a 100% match (0 single-nucleotide polymorphisms [SNPs] difference). In wave 2, a total of 5 isolates from persons in 5 separate households matched each other (0–1 SNPs difference). The 2 distinct subtypes differed by 1,178 SNPs. Of the 12 with WGS, we uploaded 10 to GenBank (accession nos. PDT001144782.1, PDT001144787.1, PDT001144788.1, PDT001161287.1, PDT001161298.1, PDT001161283.1, PDT001161285.1, PDT001166739.1, PDT001166745.1, and PDT001175404.1).

### Environmental Investigation Results

Municipal water in the town is supplied by 2 wells, each 170 feet deep. As permitted by the EPA Ground Water Rule, ground water from those wells was not treated before storage and distribution (*13*). In the 3 years preceding the outbreak, routine monthly coliform testing was positive on 5 occasions (range 1–3 instances/year). Regulatory coliform testing was negative on August 17, 2021, just before the outbreak, and on September 14, while wave 1 of the outbreak was occurring. Routine maintenance chlorination of the system, a common public water system practice typically conducted twice yearly in spring and fall, was begun by the town water operator on October 1. While the municipal water system was evaluated, chlorination of the system was continued. When chlorination was stopped on October 22, postchlorination testing detected coliform in the distribution system and in 1 source water well. Coliform detection and associated outbreak epidemiologic findings resulted in concern about possible *Campylobacter* contamination of the water supply. The town has 2 active wells located on either end of a recreation field area, a centrally located water tower, and a distribution system with 2 loops, 1 serving the bulk of the town and 1 serving newer buildings in the western part of the town. All 6 DEUF samples from the distribution system were culture-negative for *Campylobacter*. Of those, 1 sample was positive by PCR for presence of *Campylobacter* DNA. We did not detect human and bovine molecular fecal markers, but we did detect avian-specific fecal rRNA genomic material in 1 sample that also had low levels of culturable coliforms (0.72 most probable number/100 mL). This finding, together with the detected *Campylobacter* DNA, suggested the water contamination likely came from bird feces.

The most likely place for bird feces intrusion on the water system was the single, aged water tower (dating to the 1910s) pressurizing the municipal water system. Inspection of the tower by drone flyover during NDEE’s initial inspection revealed no deficits. However, given increased concern after testing results, the affected town’s leadership requested visual internal water tower inspection, which was completed on December 9 by a firm specializing in water tower maintenance. The internal tower inspection revealed damage to the tower’s cap. Gaps present between the tower’s cap and sidewall were large enough to permit direct intrusion by birds or allow bird feces from the tower’s exterior to wash inside. The town’s water was chlorinated until the tower was repaired January 28, 2022. No additional cases of campylobacteriosis were identified after chlorination of the water system was initiated while awaiting tower repair, and no campylobacteriosis clusters among community members were identified since tower repair was completed (at which time chlorination was stopped) through 1 year after tower repair completion (January 2023). In the affected community in the year after tower repair, only 1 person tested positive for *Campylobacter* by PCR. No isolate was obtained for typing because sequencing is not routinely completed in Nebraska for sporadic *Campylobacter* detections.

## Discussion

We report a large campylobacteriosis outbreak in Nebraska in which ≈19% of the town’s population reported as ill (64/≈330). In comparison, the next largest outbreak, in 2017, had 39 ill persons identified, which represented ≈6.5% of the city’s population (*31*). Identifying the outbreak cause required the combination of epidemiologic, engineering, and environmental laboratory methods. MST is an emerging surface-water quality-monitoring technique that has recently been used to support outbreak response (*32*). This investigation demonstrates the value of this combination of methods, which was essential to identify the probable source and specific site of fecal intrusion in the water system, enabling corrective action to be taken. By implicating bird contamination (probably on 2 separate occasions as suggested by WGS), our findings provided clear motivation to conduct a timely internal water tower inspection, which is technically difficult and costly.

Nebraska has a plentiful ground water supply from the High Plains Aquifer, which underlies ≈90% of the state (*12*). In total, 88% of all Nebraska residents drink ground water, and although the larger municipal water systems in the state disinfect, ≈85% of Nebraska’s 550 public ground water systems, predominantly in smaller communities, are not disinfected, (*12*,*33*). Although many states have abundant ground water from aquifers, contamination of water in the aquifer itself was never in question. Rather, aging water system infrastructure, as is often found in small or rural communities, can increase risk for enteric disease outbreaks, independent of the water’s actual source. Distribution system aging or disruption of the system during repairs can enable microbial intrusion into untreated drinking water systems (*34*). 

Water towers are a common method of water storage and pressurization in Nebraska (*35*). Rural areas, especially, are more likely to have aging water towers, sometimes more than a century old. Similar large outbreaks of enteric disease have been linked to water towers, such as a 1993 outbreak in Missouri that involved >650 cases of salmonellosis, linked to a water tower with an uncovered hatch, enabling possible entry by birds (*36*). The compromised water tower that led to this campylobacteriosis outbreak was inspected and maintained (per Title 179 NAC 21-008 and 22-008, which states that water systems must “inspect, and clean if necessary, water storage facilities equipped for accessibility, no less often than once every 5 years”) (*37*), but routine maintenance either did not identify the cap damage or the changes occurred between instances of maintenance. Our findings might indicate a need for managers of aged systems to increase scrutiny on system components and help ensure the safety of the water delivered to their residents. This need is nationwide, as demonstrated by a 2021 US infrastructure bill that has committed to “invest in water infrastructure” and particularly emphasizes this need in rural areas served by untreated ground water drinking systems (*38*).

One limitation of our study is that water testing was not undertaken until after the water system was chlorinated, 5–10 weeks after the 2 most likely periods of exposure. The inability to culture *Campylobacter* from the water samples might have been the result of previous chlorine treatment of the system or might simply reflect the common difficulty of growing *Campylobacter* in the laboratory. Had water testing been completed sooner, culturable organisms might have been found, which would have enabled genomic comparison of environmental contamination and clinical infections. In addition, selection bias probably occurred because of our use of convenience sampling. Median age of town residents was 36 years, but our questionnaire respondents’ median age was 56 years, so our respondents skewed older. Questionnaire distribution through Facebook could have created bias toward users of that social network and persons motivated to complete the questionnaires because they had been sick. However, door-to-door canvassing efforts probably helped mitigate this potential bias, given that sick and healthy persons might have been encountered equally when canvassing the town as a whole. Furthermore, given such a high proportion of town residents who used municipal water, we would not expect our results to be substantially affected based on selection. Moreover, recall bias might have occurred because questionnaire forms relied on detailed recollection of exposures potentially as long as 6 weeks after the likely exposure period. However, because the outbreak source was not a food item, respondents’ knowledge of their water source and recollection of water consumption presumably would be more reliable.

The marriage of shoe-leather epidemiology and advanced and emerging environmental microbiologic methods, with cooperation between community, local, state, and federal partners, helped establish the etiology of this large campylobacteriosis outbreak. After exposure at highly attended community gatherings was ruled out, municipal water was suspected as the cause, and then temporary disinfection and strategic investigation of the water distribution system further established the probable source of contamination. This investigation also highlights the usefulness of MST to support mitigation strategies directed at the source of fecal contamination of water systems and, in turn, to prevent future exposures. Untreated ground water systems with aging infrastructure are vulnerable to fecal intrusion, increasing the risk for large outbreaks of enteric disease. Public health authorities can encourage communities and managers of aged water systems to increase scrutiny of system components to help ensure drinking water safety.

Appendix Additional information about campylobacteriosis outbreak linked to municipal water, Nebraska, USA, 2021.
