# Supplementary material for: Campylobacteriosis Outbreak Linked to Municipal Water, Nebraska, USA, 2021
Source: Emerg Infect Dis. 2024 Oct;30(10):1998–2005. doi: 10.3201/eid3010.231509 (PMC11431921; doi:10.3201/eid3010.231509)
Supplement: Appendix — Additional information about campylobacteriosis outbreak linked to municipal water, Nebraska, USA, 2021. [file 23-1509-Techapp-s1.pdf]

Article DOI: https://doi.org/10.3201/eid3010.231509

EID cannot ensure accessibility for supplementary materials supplied by authors. Readers who have difficulty accessing supplementary content should contact the authors for assistance.

Campylobacteriosis Outbreak Linked to Municipal Water, Nebraska, USA, 2021

Appendix

Appendix Table 1. Environmental results from the affected town's water distribution system including physical and chemical water quality and biologic tests, all collected November 4, 2021.*

Sample	Water Quality Parameters[1]	Chlorine Residuals (ppm)[2]	Total Coliforms / *E. coli* (MPN/100 ml)	Somatic / F+ Coliphage (PFU/100 ml)	*Campylobacter* Result[3]	Microbial Source Tracking Result[4]
#1 Distribution system 165 L	Temperature = 17.0° C; pH = 7.30; Turbidity = 0.16 NTU; Conductivity = 597; TDS = 423 mg/l; Salinity = 0.30 ppt	Total <0.02; Free <0.02	0.72 / <0.06	<0.04 / <0.04	PCR: DNA Detected; Culture Negative	Human: Not Detected (ND); Ruminant: ND; Bird: Detected
#2 Distribution system 114 L	Temperature = 16.7° C; pH = 7.30; Turbidity = 0.07 NTU; Conductivity = 595 µS; TDS = 423 mg/l; Salinity = 0.30 ppt	Total = 0.03; Free <0.02	0.26 / <0.08	<0.06 / <0.06	PCR: ND; Culture Negative	Human: ND; Ruminant: ND; Bird: ND
#3 Distribution system 116 L	Temperature = 14.4° C; pH = 7.40; Turbidity = 0.12 NTU; Conductivity = 618 µS; TDS = 438 mg/l; Salinity = 0.30 ppt	Total <0.02; Free <0.02	0.26 / 0.08	<0.06 / <0.06	PCR: ND; Culture Negative	Human: ND; Ruminant: ND; Bird: ND
#4 Well 1 113 L	Temperature = 14.3° C; pH = 7.43; Turbidity = 0.08 NTU; Conductivity = 361 µS; TDS = 256 mg/l; Salinity = 0.20 ppt	Total <0.02; Free <0.02	<0.08 / <0.08	<0.06 / <0.06	PCR: ND; Culture Negative	Human: ND; Ruminant: ND; Bird: ND
#5 Distribution system 106 L	Temperature = 16.8° C; pH = 7.24; Turbidity = 0.05 NTU; Conductivity = 427 µS; TDS = 546 mg/l; Salinity = 0.30 ppt	Total <0.02; Free <0.02	0.09 / <0.09	<0.07 / <0.07	PCR: ND; Culture Negative	Human: ND; Ruminant: ND; Bird: ND
#6 Well 2 101 L	Temperature = 14.3° C; pH = 7.27; Turbidity = 0.08 NTU; Conductivity = 675 µS; TDS = 526 mg/l; Salinity = 0.30 ppt	Total <0.02; Free <0.02	<0.09 / <0.09	<0.07 / <0.07	PCR: ND; Culture Negative	Human: ND; Ruminant: ND; Bird: ND

Sample	Water Quality Parameters[1]	Chlorine Residuals (ppm)[2]	Total Coliforms / *E. coli* (MPN/100 ml)	Somatic / F+ Coliphage (PFU/100 ml)	*Campylobacter* Result[3]	Microbial Source Tracking Result[4]
Case Patient Home Refrigerator Filter[5]	Not Applicable (NA)	NA	ND	NA	PCR: ND; Culture Negative	Human: ND; Ruminant: ND; Bird: ND

*The results presented are generated from research-based environmental field and laboratory methods and are not intended for regulatory, clinical, or diagnostic purposes. Appropriate quality control measures were conducted for each assay and determined satisfactory. For further detail, please contact Centers for Disease Control and Prevention Waterborne Disease Prevention Branch Environmental Microbiology and Engineering Laboratory.

[1]Temperature, pH, salinity, conductivity, and total dissolved solids (TDS) measured using an Oakton 50 Multiparameter Pocket Tester. Turbidity was measured in nephelometric turbidity units (NTU) using a Hach 2100P Turbidity Meter.

[2]Disinfectant residuals were measured using a Hach Pocket Colorimeter. The total and free chlorine detection limits are 0.02.

[3]Testing included direct real time polymerase chain reaction (PCR) for *Campylobacter* using methods described by Lund et al. (2004). Detection of Campylobacter spp. in Chicken Fecal Samples by Real-Time PCR. *Journal of Clinical Microbiology*. 42, 5125–5132., followed by culturing using ISO Method 17995:2019(E): Water quality – Detection and enumeration of thermotolerant *Campylobacter* spp. *International Organization for Standardization*. ISO, Geneva, Switzerland. For culture methods, water samples were enriched in Bolton and Preston enrichment broths, followed by plating on modified charcoal cefoperazone deoxycholate agar (mCCDA) and Brain Heart Infusion Agar + 5% Rabbit Blood.

[4]Microbial source tracking assays for 1) human-, 2) ruminant-, and 3) bird-specific fecal source tracking markers were conducted following methods described in: 1) Green, H. C. et al. (2014). Improved HF183 quantitative real-time PCR assay for characterization of human fecal pollution in ambient surface water samples. I, 80(10), 3086–3094, 2) Mieszkin, S. et al. (2010). Phylogenetic analysis of *Bacteroidales* 16S rRNA gene sequences from human and animal effluents and assessment of ruminant fecal pollution by real-time PCR. *Journal of Applied Microbiology*, 108(3), 974–984; and 3) Weller, D. et al. (2020). Landscape, water quality, and weather factors associated with an increased likelihood of foodborne pathogen contamination of New York streams used to source water for produce production. *Frontiers in Sustainable Food Systems*, 3, 124, respectively.

[5]Refrigerator filter was collected from a case home who reported drinking from the filtered water during the campylobacteriosis exposure period and no further use of the filter afterward, indicating the filter would not have been subject to disinfection during the system wide periods of chlorination. The filter was processed by flushing following methods described in Mull, B., & Hill, V. R. (2012). Recovery of diverse microbes in high turbidity surface water samples using dead-end ultrafiltration. *Journal of Microbiological Methods*, 91(3), 429–433, with bacteria-specific modifications to the elution buffer composition (without sodium polyphosphate). Fabric material surrounding the internal solid carbon core filter was also processed following methods for processing a sponge swab as described in Rose, L. J. et al. (2011). National validation study of a cellulose sponge wipe-processing method for use after sampling *Bacillus anthracis* spores from surfaces. *Applied and Environmental Microbiology*, 77(23), 8355–8359.

ND: Not detected; NA: Not applicable.

Appendix Table 2. Cluster 1. Potential exposures reported by survey respondents included for analysis in a communitywide campylobacteriosis investigation and corresponding odds ratios — Nebraska, August 30–October 8, 2021.*

	No. (%)							
	Cases			Control Subjects				
Exposure**	Exposed	Not Exposed	Subtotal	Exposed	Not Exposed	Subtotal	Total	OR (95% CI)
Water	48 (98)	1 (2)	49	25 (76)	8 (24)	33	82	15.36 (1.82–129.82)
Eggs (Any)	27 (90)	3 (10)	30	9 (82)	2 (18)	11	41	2.00 (0.29–13.94)
Ground beef	24 (65)	13 (35)	37	20 (61)	13 (39)	33	70	1.29 (0.45–3.17)
Potatoes	21 (72)	8 (28)	29	9 (82)	2 (18)	11	40	0.58 (0.10–3.31)
Iceberg lettuce	19 (63)	11 (37)	30	7 (64)	4 (36)	11	41	0.99 (0.23–4.15)
Watermelon	16 (52)	15 (48)	31	5 (45)	6 (55)	11	42	1.28 (0.32–5.09)
Sweet corn	14 (50)	14 (50)	28	4 (36)	7 (64)	11	39	1.75 (0.42–7.35)
Fresh tomatoes	14 (47)	16 (53)	30	6 (55)	5 (45)	11	41	0.73 (0.18–2.92)
Raw onions	12 (40)	18 (60)	30	7 (64)	4 (36)	11	41	0.38 (0.09–1.59)
Cantaloupe	12 (38)	20 (63)	32	7 (64)	4 (36)	11	43	0.34 (0.09–1.42)
Cucumbers	11 (72)	18 (28)	29	5 (45)	6 (55)	10	39	0.61 (0.14–2.60)
Apples	11 (37)	19 (63)	30	4 (36)	7 (64)	11	41	1.01 (0.24–4.26)
Carrots	10 (34)	19 (66)	29	3 (27)	8 (73)	11	40	1.40 (0.30–6.49)
Mini carrots	10 (34)	19 (66)	29	4 (36)	7 (64)	11	40	0.92 (0.22–3.92)
Sweet or bell peppers	10 (34)	19 (66)	29	5 (45)	6 (55)	11	40	0.63 (0.15–2.59)
Pork	10 (27)	27 (73)	37	16 (50)	16 (50)	32	69	0.37 (0.14–1.01)
Grapes	9 (31)	20 (69)	29	6 (55)	5 (45)	11	40	0.38 (0.09–1.56)
Poultry	8 (21)	30 (79)	38	14 (42)	19 (58)	33	71	0.36 (0.13–1.03)
Gathering	8 (21)	30 (79)	38	14 (45)	17 (55)	31	69	0.32 (0.11–0.93)
Eggs (farm fresh)	7 (35)	13 (65)	20	3 (50)	3 (50)	6	26	0.54 (0.09–3.41)
Peaches or nectarines	7 (23)	23 (77)	30	4 (36)	7 (64)	11	41	0.53 (0.12–2.37)
Fresh salsa	6 (20)	24 (80)	30	2 (18)	9 (82)	11	41	1.13 (0.19–6.63)
Broccoli or cauliflower	5 (17)	24 (83)	29	2 (18)	9 (82)	11	40	0.94 (0.15–5.73)
Strawberries	5 (17)	24 (83)	29	6 (55)	5 (45)	11	40	0.17 (0.04–0.80)
Pears	5 (17)	25 (83)	30	0	11 (100)	11	41	–
Celery	4 (14)	25 (86)	29	5 (50)	5 (50)	10	39	0.16 (0.03–0.82)
Hot peppers	3 (10)	26 (90)	29	2 (18)	9 (82)	11	40	0.52 (0.07–3.62)
Cabbage	3 (10)	26 (90)	29	2 (20)	8 (80)	10	39	0.46 (0.07–3.27)
Raw eggs	3 (10)	26 (90)	29	4 (36)	7 (64)	11	40	0.20 (0.04–1.12)

	No. (%)							
	Cases			Control Subjects				
Exposure**	Exposed	Not Exposed	Subtotal	Exposed	Not Exposed	Subtotal	Total	OR (95% CI)
Romaine lettuce	3 (10)	27 (90)	30	2 (18)	9 (82)	11	41	0.50 (0.07–3.49)
Green beans	3 (10)	27 (90)	30	4 (36)	7 (64)	11	41	0.19 (0.04–1.08)
Other raw roots and vegetables	2 (7)	27 (93)	29	1 (9)	10 (91)	11	40	0.74 (0.06– 9.09)
Blueberries	2 (7)	27 (93)	29	2 (18)	9 (82)	11	40	0.34 (0.04–2.72)
Zucchini or summer Squash	2 (7)	27 (93)	29	2 (18)	9 (82)	11	40	0.33 (0.04–2.72)
Spinach	2 (7)	28 (93)	30	2 (18)	9 (82)	11	41	0.32 (0.04–2.62)
Green onions or scallions	2 (7)	28 (93)	30	3 (30)	7 (70)	10	40	0.17 (0.02–1.20)
Oranges	2 (7)	28 (93)	30	4 (36)	7 (64)	11	41	0.13 (0.02–0.83)
Eggplant	1 (3)	28 (97)	29	2 (18)	9 (82)	11	40	0.16 (0.01–1.99)
Cherries	1 (3)	28 (97)	29	0	11 (100)	11	40	–
Melons (other)	1 (3)	29 (97)	30	1 (9)	10 (91)	11	41	0.34 (0.02–6.04)
Animal contact***	20 (45)	24 (55)	44	22 (67)	11 (33)	33	77	0.42 (0.16–1.06)
Manure applied to garden***	0	27 (100)	27	0	11 (100)	11	38	–

* Individuals answering “Yes” and “Maybe” to exposure were counted as “Exposed.” Individuals answering “Don't Know” were excluded from analysis. Percentages have been rounded to the nearest whole number. Percentages might not sum to 100 because of rounding.
** Table excludes *** exposures in which ill individuals were queried but none reported exposure across first and second wave
*** Exposure not necessarily associated with consumption of a specific food or drink item.

Appendix Table 3. Cluster 2. Potential exposures reported by survey respondents included for analysis in a communitywide campylobacteriosis investigation and corresponding odds ratios — Nebraska, August 30–October 8, 2021.*

	No. (%)							
	Cases			Control Subjects				
Exposure**	Exposed	Not Exposed	Subtotal	Exposed	Not Exposed	Subtotal	Total	OR (95% CI)
Water	14 (100)	0	14	22 (65)	12 (35)	34	48	16.11 (0.88–293.6)****
Ground beef	11 (100)	0	11	26 (81)	6 (18)	32	43	5.64 (0.29–108.7)****
Iceberg lettuce	8 (80)	2 (20)	10	17 (59)	12 (41)	29	39	2.82 (0.51–15.72)
Fresh tomatoes	8 (80)	2 (20)	10	20 (69)	9 (31)	29	39	1.80 (0.32–10.23)
Eggs (any)	8 (80)	2 (20)	10	24 (83)	5 (17)	29	39	0.83 (0.13–5.17)
Potatoes	7 (78)	2 (22)	9	21 (75)	7 (25)	28	37	1.17 (0.19–6.98)
Watermelon	7 (70)	3 (30)	10	13 (45)	16 (55)	29	39	2.87 (0.62–13.37)
Cucumbers	6 (60)	4 (40)	10	22 (76)	7 (24)	29	39	0.48 (0.10–2.19)
Zucchini and summer squash	5(50)	5(50)	10	10 (34)	19 (66)	29	39	1.90 (0.44–8.15)
Eggs (farm fresh)	5 (63)	3 (38)	8	10 (43)	13 (57)	23	31	2.17 (0.42–11.30)
Raw onion	5 (50)	5 (50)	10	15 (56)	12 (44)	27	37	0.80 (0.19–3.42)
Pork	5 (45)	6 (55)	11	17 (53)	15 (47)	32	43	0.74 (0.19–2.91)
Tomato (on salad or burgers)	4 (40)	6 (60)	10	6 (23)	20 (77)	26	36	2.22 (0.47–10.57)
Mini carrots	4 (40)	6 (60)	10	8 (28)	21 (72)	29	39	1.75 (0.39–7.88)
Cantaloupe	4 (40)	6 (60)	10	10 (34)	19 (66)	29	39	1.27 (0.29–5.56)
Cabbage	4 (40)	6 (60)	10	10 (36)	18 (64)	28	38	1.20 (0.27–5.29)
Grapes	4 (40)	6 (60)	10	14(50)	14 (50.0)	28	38	0.67 (0.15–2.89)
Sweet or bell peppers	4 (40)	6 (60)	10	14 (50)	14 (50.0)	28	38	0.67 (0.15–2.89)
Apples	4 (40)	6 (60)	10	15 (52)	14 (48)	29	39	0.62 (0.14–2.68)
Broccoli	3 (30)	7 (70)	10	7 (24)	22 (76)	29	39	1.35 (0.27–6.66)
Carrots	3 (30)	7 (70)	10	14 (48)	15 (52)	29	39	0.46 (0.10–2.13)
Sweet corn	3 (30)	7 (70)	10	21 (75)	7 (25)	28	38	0.14 (0.03–0.71)
Poultry	3 (27)	8 (73)	11	20 (63)	12 (38)	32	43	0.23 (0.05–1.02)
Romaine lettuce	2 (20)	8 (80)	10	3 (11)	25 (89)	28	38	2.08 (0.29–14.77)
Hot Peppers	2 (20)	8 (80)	10	8 (28)	21 (72)	29	39	0.66 (0.11–3.78)
Pears	2 (20)	8 (80)	10	8 (28)	21 (72)	29	39	0.66 (0.11–3.78)
Gathering	2 (20)	8 (80)	10	12 (38)	20 (63)	32	42	0.42 (0.08–2.30)
Green beans	2 (20)	8 (80)	10	11 (39)	17 (61)	28	38	0.39 (0.07–2.17)
Celery	2 (20)	8 (80)	10	12 (41)	17 (59)	29	39	0.35 (0.06–1.97)
Raw egg	1 (10)	9 (90)	10	1 (3)	28 (97)	29	39	3.11 (0.18–54.97)

	No. (%)							
	Cases			Control Subjects				
Exposure**	Exposed	Not Exposed	Subtotal	Exposed	Not Exposed	Subtotal	Total	OR (95% CI)
Blueberries	1 (10)	9 (90)	10	2 (7)	27 (93)	29	39	1.50 (0.12–18.57)
Spinach	1 (10)	9 (90)	10	3 (11)	25 (89)	28	38	0.93 (0.09–10.09)
Raw green onion or scallion	1 (10)	9 (90)	10	4 (14)	25 (86)	29	39	0.69 (0.07–7.07)
Cherries	1 (10)	9 (90)	10	4 (14)	24 (86)	28	38	0.67 (0.07–6.79)
Fresh salsa	1 (10)	9 (90)	10	4 (15)	23 (85)	27	37	0.64 (0.06–6.52)
Peaches or nectarines	1 (10)	9 (90)	10	7 (25)	21 (75)	28	38	0.33 (0.04–3.12)
Strawberries	1 (10)	9 (90)	10	8 (28)	21 (72)	29	39	0.29 (0.03–2.69)
Eggplant	0	10 (100)	10	3 (10)	26 (90)	29	39	–
Animal contact***	6 (50)	6 (50)	12	10 (31)	22 (69)	32	44	2.20 (0.56–8.54)
Manure applied to garden***	1 (11)	8 (89)	9	1 (4)	24 (96)	25	34	3.00 (0.17–53.71)

* Individuals answering “Yes” and “Maybe” to exposure were counted as “Exposed.” Individuals answering “Don't Know” were excluded from analysis for that specific variable. Percentages have been rounded to the nearest whole number. Percentages might not sum to 100 because of rounding.

** Table excludes exposures in which no ill individuals reported exposure across first and second wave

*** Exposure not necessarily associated with consumption of a specific food or drink item

**** Odds Ratio originally calculated as undefined so the modified Haldane–Anscombe correction was applied to calculate odds ratio
